# Immune Mechanisms Underlying Hepatitis B Surface Antigen Seroclearance in Chronic Hepatitis B Patients With Viral Coinfection

**DOI:** 10.3389/fimmu.2022.893512

**Published:** 2022-05-11

**Authors:** Shuling Wu, Wei Yi, Yuanjiao Gao, Wen Deng, Xiaoyue Bi, Yanjie Lin, Liu Yang, Yao Lu, Ruyu Liu, Min Chang, Ge Shen, Leiping Hu, Lu Zhang, Minghui Li, Yao Xie

**Affiliations:** ^1^ Department of Hepatology Division 2, Beijing Ditan Hospital, Capital Medical University, Beijing, China; ^2^ Department of Gynecology and Obstetrics, Beijing Ditan Hospital, Capital Medical University, Beijing, China; ^3^ Department of Hepatology Division 2, Peking University Ditan Teaching Hospital, Beijing, China

**Keywords:** hepatitis B virus, hepatitis B surface antigen, functional cure, coinfection, immune

## Abstract

It is considered that chronic hepatitis B patients have obtained functional cure if they get hepatitis B surface antigen (HBsAg) seroclearance after treatment. Serum HBsAg is produced by cccDNA that is extremely difficult to clear and dslDNA that is integrated with host chromosome. High HBsAg serum level leads to failure of host immune system, which makes it unable to produce effective antiviral response required for HBsAg seroclerance. Therefore, it is very difficult to achieve functional cure, and fewer than 1% of chronic hepatitis B patients are cured with antiviral treatment annually. Some chronic hepatitis B patients are coinfected with other chronic viral infections, such as HIV, HCV and HDV, which makes more difficult to cure. However, it is found that the probability of obtaining HBsAg seroclearance in patients with coinfection is higher than that in patients with HBV monoinfection, especially in patients with HBV/HIV coinfection who have an up to 36% of HBsAg 5-year-seroclerance rate. The mechanism of this interesting phenomenon is related to the functional reconstruction of immune system after antiretroviral therapy (ART). The quantity increase and function recovery of HBV specific T cells and B cells, and the higher level of cytokines and chemokines such as IP-10, GM-CSF, promote HBsAg seroclearance. This review summarizes recent studies on the immune factors that have influence on HBsAg seroconversion in the chronic hepatitis B patients with viral coinfection, which might provide new insights for the development of therapeutic approaches to partially restore the specific immune response to HBV and other viruses.

## Introduction

There are more than 250 million hepatitis B virus (HBV) carriers in the world, and about 600 000 patients die of HBV-related liver diseases every year (1, 2). The pathogenesis of hepatitis B is considered to be related to the host immune response, but the underlying mechanism is not completely clear at present. In the immune tolerance state, the virus replicates a lot, and the levels of serum hepatitis B surface antigen (HBsAg) and hepatitis B e antigen (HBeAg) are very high. After entering the stage of immune clearance, the virus replication decreases, and the levels of HBsAg and HBeAg decrease as well. HBV DNA can be inhibited by effective antiviral treatment, but it is hard to clear covalently closed circular DNA (cccDNA) and double stranded linear DNA (dslDNA) which integrated with the host chromosome. As a result, it is quite difficult to completely clear serum HBsAg. Clearance of serum HBsAg with or without anti-HBs is defined as hepatitis B functional cure (3, 4). In order to improve the rate of functional cure, some new drugs have been developed and entered the stage of clinical trials. In the long process of chronic hepatitis B virus infection, the human body may also be coinfected with other hepatophilic or non-hepatophilic viruses. These viruses inhibit or activate human immunity, making the immune clearance mechanism of HBsAg more complex.

## Three Forms of HBSAG Derived From Two Sources

**Figure 1:**
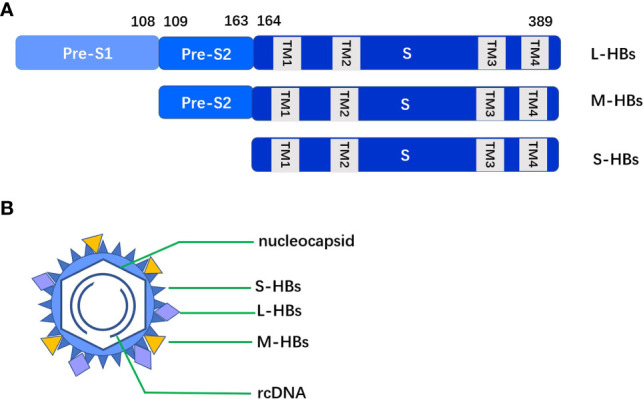
**(A)** The three S proteins (L-HBs, M-HBs and S-HBs) differ in their N- terminus but share a common S domain with 4 putative TM domains on their C-terminus. **(B)** Intact HBV particles contain a large amount of S-HBs and the same amount of M-HBs and L-HBs, with a composition ratio of about 4:1:1.

The total length of HBV genome is about 3.2 kb, containing four partially or completely overlapping open reading frames (ORF) C, S, P and X. HBsAg is encoded by the S ORF, which contains PreS1, PreS2 and S. The production of HBsAg comes mainly from cccDNA. The 2.4 kb and 2.1 kb S mRNA of cccDNA transcripts, that is PreS1/S and PreS2/S respectively, then translates into three sizes of proteins: L-HBs (PreS1+PreS2+S), M-HBs (PreS2+S), and S-HBs (S). The three S proteins differ in their N- terminus but share a common S domain with 4 putative transmembrane (TM) domains on their C-terminus. PreS1 has 108-109 amino acid residues, PreS2 has 55 amino acid residues and S has 226 amino acid residues (see **Figure 1**).

**Figure 2:**
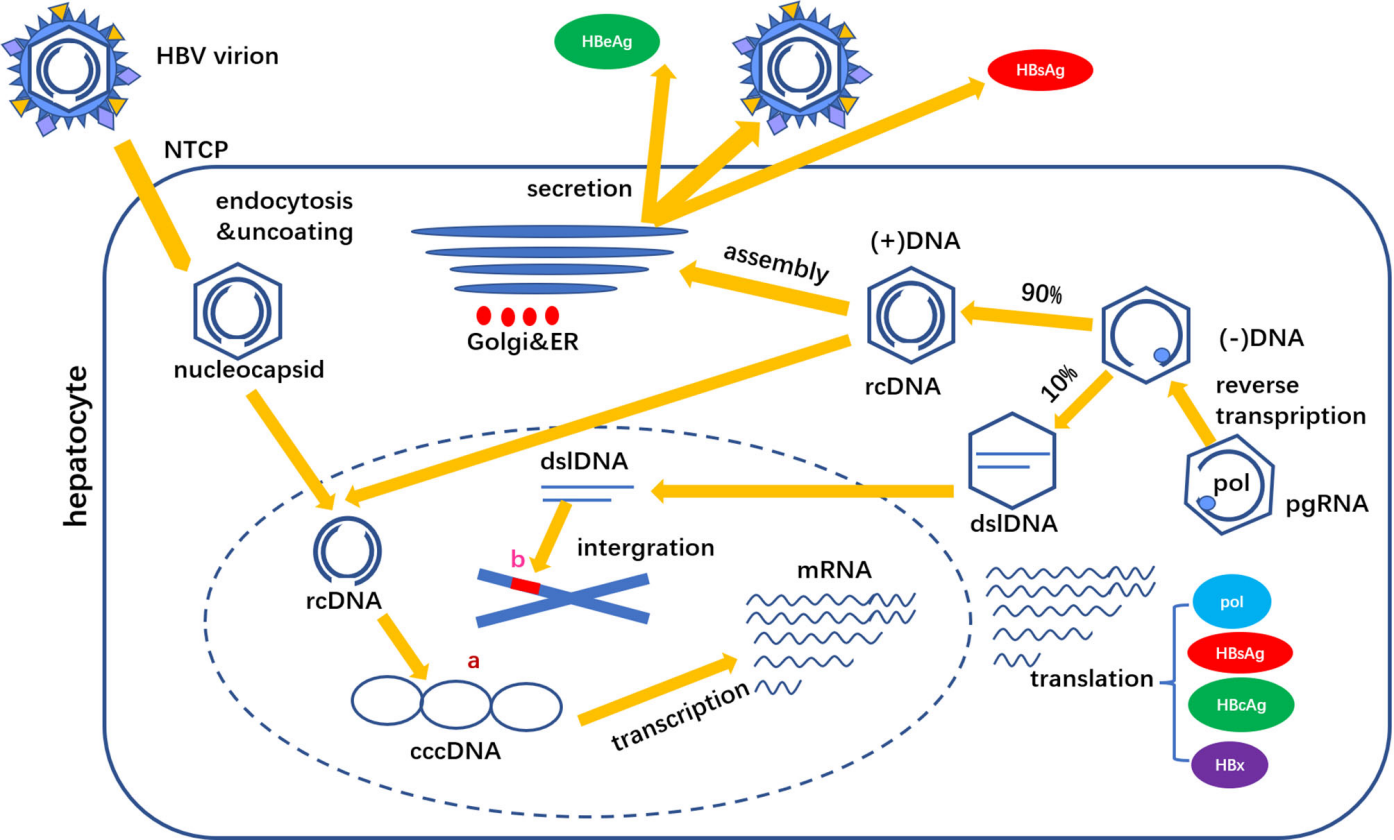
HBsAg is derived from two sources **(A)** cccDNA and **(B)** dslDNA. cccDNA is the main source of HBsAg production.

Another source of HBsAg is dslDNA integrated with host genes. The cccDNA transcript pregenomic RNA (pgRNA) is reverse transcribed into negative strand DNA, of which about 90% is synthesized relaxed circular DNA (rcDNA) and about 10% is synthesized dslDNA. After shelling, dslDNA enters the hepatocyte nucleus and integrates in the host gene chromosome. This integration can occur in the early stage of HBV infection, but the integration level in HBeAg positive stage is low, and the integration in HBeAg negative stage is frequent (5). HBsAg production pathways are shown in **Figure 2**.

The integrated DNA is no longer involved in the formation of virus particles, but can translate into HBsAg. Due to the deletion of some PreS in dslDNA, the proportion of M-HBs and L-HBs in HBsAg from integrated HBV DNA is low. HBsAg translated through the above two pathways accumulates in the endoplasmic reticulum (ER) and forms agglomerates with different cysteines in the S region through covalent disulfide bonds. Intact HBV particles contain a large amount of S-HBs and the same amount of M-HBs and L-HBs, with a composition ratio of about 4:1:1 (6). HBsAg in virus particles accounts for about 1/3 of the total amount of HBsAg, other HBsAg exists in small spherical subvirus particles and filamentous particles.

A recent study found that the proportion of M-HBs in HBeAg positive patients is the best predictor of early HBsAg clearance before nucleoside analogue (NA) treatment. The median level of M-HBs in patients with HBsAg clearance before treatment is significantly lower than that in patients without HBsAg clearance. The proportion of M-HBs and L-HBs decreases rapidly during treatment, and M-HBs cannot be detected after half a year of treatment. In patients with HBsAg clearance treated with pegylated interferon (PEG-IFN), the proportion of M-HBs and L-HBs also shows similar dynamic changes (7). The mechanisms underlying the change in HBsAg composition prior to HBsAg loss is unknown. It is assumed that the structural arrangement of the integrated dslDNA form does not necessarily affect the expression of S-HBs, but parts of PreS may be missing. HBsAg derived from integrated dslDNA contains a low proportion of M-HBs and L-HBs. M-HBs and L-HBs mainly derived from cccDNA. Consequently, the decrease in M-HBs and L-HBs before HBsAg loss might reflect a progressive shutdown of cccDNA activity, but more research is needed to verify this hypothesis.

## HBSAG is Related to HBV Specific Immune Dysfunction

After human body is infected by HBV, the virus is jointly cleared by innate and specific immune responses. However, the simultaneous nonspecific immune response will cause liver inflammation and necrosis, and even occurrence of liver cirrhosis and liver cancer (8). The immune function of patients with chronic hepatitis B (CHB) is impaired. On the one hand, the immune function of HBV specific T cells is low. such as the increase of immune negative regulatory components [regulatory T cells (Treg), myeloid derived suppressor cells (MDSC), programmed death receptor 1 (PD1), transforming growth factor (TGF-β), interleukin (IL)-10, *etc*], depressed effector function and proliferation ability, imbalance of cytokine network. As a result, the body is unable to eliminate effectively the virus, leading to continuous replication of HBV in the human body.

On the other hand, the immune response of non-HBV specific CD8^+^ T cells, natural killer (NK) cells and T helper (Th) 17 cells is enhanced, which can cause liver damage. A large number of non-HBV specific CD8^+^T cells infiltrate in the liver of patients with CHB, and their ability to proliferate and produce IL-2 is significantly reduced, but the function of other proinflammatory factors is not damaged, such as interferon (IFN)-γ and tumor necrosis factor (TNF)- α, resulting in nonspecific inflammatory injury (9, 10).

For those HBeAg positive patients in immune activation stage, there is a significant increase in NK cell activity because of increased expression of IL-12, IL-15 and IL-18 in liver and decreased expression of IL-10, NK cells enhance the killing ability, but the ability of secreting IFN-γ is not enhanced, causing liver damage but not clearance of virus. In addition, NK cells can also mediate hepatocyte apoptosis through the upregulation expression of TNF related apoptosis inducing ligand (TRAIL). A large number of Th17 cells infiltrate in the liver of patients with CHB, and is positively correlated with viral load (HBV DNA), alanine aminotransferase (ALT) level and histological activity. IL-17 secreted by Th17 cells mainly promotes the secretion of IL-1β, IL-6, TNF-α and IL-23 inflammatory factors by myeloid dendritic cells (MDC) and monocytes to mediate liver injury (11, 12).

What role does HBsAg play in immune disorder in patients with chronic hepatitis B? It is found that a large amount of HBsAg is the main factor associated with low anti HBV specific immune function. Although there is no strong evidence to support that HBsAg can directly inhibit HBV specific immune response, some studies suggest that HBsAg is related to HBV specific immune dysfunction (13, 14). High level of HBsAg is associated with the impairment of anti-HBV specific T and B cell immune function. Reducing the HBsAg level should promote the recovery of specific immune function, and in turn promote the clearance of HBsAg. When HBsAg clearance or HBsAg seroconversion is realized, the anti-HBV immune response function of the body nearly returns to normal. The incidence of HBsAg variation increases when there is an excessive immune response, such as slow plus acute liver failure (15). HBsAg can inhibit monocyte activity by binding to specific receptors on monocytes, and HBsAg can cause dysfunction of MDC and plasma cell like dendritic cells (PDC). HBsAg can also increase the response of IL-23/IL-17 axis and mediate liver immune injury (16).

### HBsAg and Innate Immunity

Innate immunity is the first defense line against microbial infection, which relies on different pattern recognition receptors (PRRS) to recognize nucleic acids. Hepatitis B virus infection can activate inflammatory factors through two main types of PRR i.e., Toll like receptor (TLR) signal pathway (17–19), and retinoic acid induced gene 1 (RIG-1) signaling pathway (19–21). The expression levels of TLR3, RIG-1 and melanoma differentiation associated gene 5 (MDA5) in peripheral blood of patients with chronic hepatitis B are significantly decreased, which may account for the chronic state of HBV infection. HBsAg can inhibit innate immunity by inhibiting TLR-mediated signaling pathway and inducing IL-10 in Kupffer cells (KCS) and sinusoidal endothelial cells (LSEC) (17). In the presence of HBsAg, the function of myeloid dendritic cells (MDC) is also impaired, which stimulates T cell response (16). In another study, DC isolated from CHB patients is functional, and DC stimulates autologous HBV specific T cell expansion through the cross presentation of circulating HBsAg (22). Most experiments of HBsAg mediated innate immunity are carried out *in vitro*, which is related to the difference of these results. A recent study shows that HBsAg suppressed the activation of the nuclear factor kappa B (NF-кB) pathway *via* interaction with the TAK1-TAB2 complex, leading to downregulation of innate immune responses (23).

### HBsAg and Cellular Immunity

Dysfunction and failure of HBV specific CD8^+^ T cell response are markers of chronic HBV infection (24, 25). High levels of HBsAg in circulation and liver may lead to impaired HBsAg specific CD8^+^ T cell response through continuous antigen stimulation. In addition, HBsAg can inhibit T cell response and enhance regulatory T cell response by promoting the differentiation of monocytes into MDSC (26). In the woodchuck hepatitis virus (WHV) transgenic mouse model, high levels of viral replication and protein expression in male mice induces the expansion of regulatory T cells in the liver, resulting in impaired WHV specific CD8^+^ T cell response and gender related differences in virus infection results (27). Compared with healthy persons, the proportion of myeloid dendritic cells (MDC) and plasma like dendritic cells (PDC) in patients with chronic hepatitis B is by and large normal, but MDC has the decreased ability of providing costimulatory signals to T cells and secreting cytokines such as TNF-α. The main function of these cytokines is to promote the maturation of DC and proliferation of DC induced T cells (28). In this process, the HBsAg and HBV DNA levels are high, suggesting that the presence of these two viral components may damage the function of MDC. Other studies have shown that in transgenic mouse models, circulating HBsAg clearance does not improve HBV specific CD8+ T cell response *in vivo* (29).

### HBsAg and Humoral Immunity

B cell response may play an important role in controlling HBV infection. For example, the clinical application of rituximab, which consumes B cells, can lead to the reactivation of HBV in controlled patients. This suggests that the response of B cells to HBV is essential for maintaining effective host immune control of HBV (30–32). In chronic hepatitis B patients, the antigen presenting function of HBsAg and the dysfunction of CD4^+^ T cells due to the high level of DC can affect the secretion of anti-HBs by HBV specific B cells. This may also lead to insufficient affinity or no function of anti-HBs, so it can’t play the role of neutralizing antibody.

HBs-ELISPOT and flow cytometry fluorescence sorting (FACS) technology were used to detect HBsAg specific memory B cells (CD19 cells) in HBV vaccine inoculation staff and CHB patients. These two methods detected a small number of HBsAg specific B cells in HBV vaccine inoculation staff, but none was detected in CHB patients (33–35). Other studies have shown that the frequency of HBsAg specific B cells in blood is similar in patients with acute, chronic and cured HBV infection, and has no relationship with the serum levels of HBsAg, HBV DNA or ALT (36, 37).

HBsAg specific B cells from patients with chronic hepatitis B are atypical B cells, characterized by low expression of CD21 and CD27, but high expression of inhibitory markers such as PD-1 and T-bet. In addition, HBsAg specific B cells from patients with chronic hepatitis B are not able to mature into anti-HBs secreting cells *in vitro*. However, their function can be partially restored by specific culture conditions, such as PD-1 blocking or adding IL-2, IL-21 and CD40L (38). Le Bert (39) et al. found that in CHB patients, HBcAg specific B cells were more frequent than HBsAg specific B cells. The phenotypic and functional differences between HBsAg and hepatitis B core antigen (HBcAg) specific B cells in a same patient suggest that high levels of HBsAg may lead to programming obstacles of HBsAg specific B cells through continuous stimulation. Follicular helper T cell can improving HBsAg-specific B cell response in chronic hepatitis B patients targeting by TLR8 signaling (40).

In some CHB patients, the presence of anti-HBs and HBsAg coexists. Only the presence of anti-HBs may eliminate HBsAg in peripheral blood, but it will not terminate chronic HBV infection in the liver. Therefore, HBsAg specific B cell response contributes to HBV pathogenesis, clearance and protective immunity, but it is not enough to control HBV infection alone (41).

## Immune Mechanisms of Current Drugs and Emerging Therapies Targeting Functional Cure of Hepatitis B

Persistent HBsAg seroclearance after treatment, and with or without anti-HBs serologic conversion, is the ideal end point of antiviral therapy for chronic hepatitis B, which represents sustained virological inhibition and immunological control. Current hepatitis B antiviral treatments include two main classes of drugs, one is the oral NAs and the other is injected IFN/PEG-IFN.

It is difficult to achieve the goal of HBsAg seroclearance with standard antiviral treatments. Functional cure occurs at an average annual rate of 0.22% in CHB patients during first-line oral NAs antiviral treatment (42). PEG-IFN treatment can acquire an average HBsAg clearance annual rate of 3% (43–45), 5-year cumulative rate of 14% and 10-year cumulative rate of 32% (46) in CHB patients. But in inactive HBsAg carriers, the HBsAg clearance rate can reach to 47% after 48 weeks of PEG-IFN treatment (47). New treatment strategies such as combination therapy (initial combination therapy of NAs and IFN/PEG-IFN, continuous combination therapy of NAs and IFN/PEG-IFN) and new therapeutic drugs may help patients improve the negative conversion rate of HBsAg and even the seroconversion rate of HBsAg.

NAs and IFN/PEG-IFN play different roles in host immune response. IFN mainly regulates innate immune response, especially NK cell activity. Micco et al. (48) found that PEG-IFN can induce the production of IL-15 and promote the activation and expansion of CD56^bright^ NK cells, so as to enhance its antiviral activity and promote IFN-γ expression of apoptosis inducing ligand related to soluble TNF. PEG-IFN may lead to the continuous consumption of effector CD8^+^ T cells, and has limited repair effect on the function of HBV specific CD8^+^ T cells. NAs cannot resume the antiviral ability of NK cells, but temporarily repair the function of damaged T lymphocytes. In patients with virological inhibition after long-term NAs treatment, the damaged function of HBV specific T lymphocytes is partially restored *in vitro* (49–55). These studies showed that NAs might promote the recovery of T cell function mainly through inhibiting HBV replication. Besides, increased NK cell function is associated with active hepatitis and HBsAg seroclearance following structured NAs cessation (56). Host immune function repair is a key step to achieve chronic hepatitis B functional cure. The rationality of the combined treatment strategy of NAs and IFN/PEG-IFN lies in that the two kinds of antiviral mechanisms play different roles in innate immunity and adaptive immunity. The inhibition of HBV replication by NAs can enhance the activation of IFN on innate immunity (57, 58).

New drugs targeting at the functional cure of hepatitis B mainly include two main categories: direct antiviral drugs and indirect antiviral drugs. The former directly targets viral diseases and interfere with the replication process of HBV DNA, and the latter targets the host immune system to attack HBV. Direct antiviral drugs include siRNA (ARC-520 and JNJ-3989) (59, 60), HBV entry inhibitor (Bulevirtide, formerly known as Myrcludex B) (61), core protein allosteric regulator (NVR 3-778, JNJ-56136379, RO7049389 and ABI-H0731) (62–65), antisense RNA (IONIS-HBVRx and IONIS-HBVLRx) (66), cccDNA inhibitor (not yet in clinical trial), HBsAg release inhibitor (REP 2139) (67), HBsAg neutralizer (lenvervimab) (68), *etc.*

Indirect antiviral drugs include Toll like receptor (TLR) agonists (vesatolimod, selgantolimod) (69), immune checkpoint inhibitors (anti-PD-L1) (70), therapeutic vaccines (GS-4774) (71), engineering T cells, *etc.* Among them, the effect of therapeutic vaccine is disappointing (72). The combination of existing and new antiHBV drugs may improve HBsAg seroclearance rate (73), and the elimination of HBV requires a treatment scheme based on a combination of multiple drugs.

## Immune Mechanisms and HBSAG Serum Level Changes in HBV Coinfection Patients

HBV infection here only refers to chronic HBV infection, that is, HBsAg positive lasts for more than half a year. HBV coinfection only includes HBV and other viruses, excluding bacteria, fungi, parasites, protozoa and other infections. According to the tropism of coinfected viruses, they can be divided into coinfected hepatophilic viruses such as hepatitis C virus (HCV), hepatitis D virus (HDV), hepatitis E virus (HEV), hepatitis A virus (HAV), and non-hepatophilic viruses such as human immunodeficiency virus (HIV).

### HBV/HAV Coinfection

HAV is often transmitted through fecal-oral route and mostly leads to acute and self-limiting infection. In a few of cases, it can cause severe liver function damage or even liver failure (74). Acute HAV infection used to occur in adolescents who were not vaccinated against hepatitis A. Due to the emergence of hepatitis A vaccine, hepatitis A has become increasingly a disease of adults in many parts of the world. The pathogenesis of acute hepatitis A tends to be dominated by host immune response, and HAV causes a weak interferon response in the liver of infected chimpanzees (75). Compared with CD8^+^ T cell response, immune control of HAV may be more directly related with CD4^+^ T cells (76). The frequency of HAV specific CD8^+^ T cells in blood and liver of patients with jaundice may decrease with the clearance of infection (77).

In Ifnar1^-/-^ transgenic mice, HAV induced hepatocyte apoptosis and inflammatory response are activated by innate immunity (78). Innate cytotoxic cells and Treg cells are transformed into inflammatory phenotypes in symptomatic infected individuals (79, 80). In HBV-infected PXB cells superinfected with HAV, HBV replication was reduced as compared to that in PXB cells infected with HBV alone, which means to a certain extent, HAV infection inhibits HBV replication (81). Earlier study also found that infection with HBV downregulated the expression of the two HBV proteins (HBsAg and PreS2) in PLC/PRF/5 cells (82). The sharp rise in IFN-γ production mediated by the acute HAV infection may be pivotal in the suppression of HBV replication in chronic hepatitis B (83). Fu et al. (84) retrospectively analyzed 211 HBV coinfection patients in a tertiary teaching hospital in China from 2005 to 2014, and 35 patients were coinfected with HAV. Patients with HAV coinfection generally had better outcomes than those with other viruses coinfection. Sagnelli et al. (85) reported that 3 of 9 patients with HBV/HAV coinfection became negative for HBsAg after 6-month follow-up. Beisel et al. (86) reported a 47-year-old patient with HBV-related compensated cirrhosis who had an acute HAV superinfection. The spontaneous HBsAg seroconversion occurred and the non-specific immunity of HAV led to functional cure of hepatitis B. Acute HAV superinfection may trigger sustained clearance of HBsAg in patients with chronic HBV infection.

### HBV/HEV Coinfection

HEV is transmitted usually through fecal-oral pathway and occasionally through blood transfusion pathway (87). HEV is also a zoonotic virus in some genotypes. Acute HEV infection generally occurs in adults. Wong et al. (88) found that the seropositive rate of antiHEV-IgG was 19.86% among HBV infected patients by using the data of the National Health and Nutrition Examination Survey from 2011 to 2018. In a cross-sectional study in Vietnam from 2012 to 2013, the seropositive rate of antiHEV-IgM was 11.6% among HBV infected patients (89).

In acute hepatitis E infection patients, the percentage of NK and NKT cells in peripheral blood monocytes decreased significantly, while the ratio of activated NK and NKT cells was higher than that in the uninfected group (90). The expression of activated NK cell markers Granzyme B and CD69 also increased significantly (91). The severe condition of pregnant women infected with HEV is related to the decrease of NK cell activity (92). In pregnant women with HEV infection, the inflammatory cytokine TNF-α, IL-6 and IFN-γ level increased significantly (93).

A large amount of evidence shows that TNF-α and NF-κB signaling pathways play an important role in stimulating inflammatory response in HEV. In cell culture, TNF-α has been shown to moderately inhibit HEV replication. Interestingly, it can cooperate with IFN-α anti HEV effect through NF-κB cascade inducing a subset of IFN-stimulated gene (ISG) (94).

HEV coinfection can accelerate the disease progression of patients with chronic HBV infection and increase the mortality of patients with liver cirrhosis. Acute HEV superinfection was associated with a 1-year mortality rate of 2.4% in non-cirrhotic patients with chronic HBV infection. The 1-year mortality rate increased to 35.7% in patients with compensated liver cirrhosis after HEV superinfection. HEV superinfection increased the long-term risk of cirrhosis, hepatocarcinoma, and liver-related death in patients with chronic HBV infection (95).

Compared with HBV monoinfection, the expression of cytokines related to hepatocyte necrosis such as IL-6, IL-10 and TNF-α increased in HBV/HEV coinfection patients (96). There are scarce and conflicting data regarding the replication of viruses in coinfection patients. The median level of HBV DNA in HBV/HEV coinfection patients is lower than that in HBV monoinfected patients. However, due to the small number of samples, it is not clear whether this difference is statistically significant. In addition, baseline HBV DNA are not available to compare with HBV DNA levels after HEV superinfection (97). The higher HBV DNA level in patients with HBV monoinfection may be explained by that HEV is an RNA virus, which may play a role of ribozyme in HBV DNA replication (96). There is no significant difference in HBV DNA levels between CHB/HAV coinfection patients and CHB/HEV coinfection patients (98). Yeh et al. (99) reported the disappearance of HBsAg in a renal transplant patient with chronic HBV/HEV coinfection. However, we cannot draw a conclusion about the effect of HEV on HBsAg from a single case, and further studies are required to evaluate this hypothesis.

### HBV/HCV Coinfection

HCV is a single stranded RNA virus, which mainly leads chronic infection. Innate immune response is very important for HCV infection. It limits virus transmission by inducing apoptosis of infected hepatocytes and stimulates antigen specific adaptive immune response. NK cells destroy infected hepatocytes and cytokine release through cytolysis, which plays a vital role in the innate immune response to acute HCV infection.

IFN produced by NK cells can directly inhibit HCV replication. IFN-γ and TNF-α lead to maturation of dendritic cells, release of IL-12 and differentiation of CD4 and CD8^+^ T cells. Specific CD8^+^ T cells destroys HCV infected hepatocytes through human leukocyte antigen (HLA) class I antigen presenting cells and induces cytokines (TNF-α and IFN-γ) secretion. Helper CD4^+^ T cells support this function through IL-2 to stimulate activation of CD8^+^ T cells and NK cells (100).

During chronic HCV infection, the production of IL-2 by HCV specific CD4+ T cells decreases, resulting in impaired activation of CD8+ T cells. HCV core protein and PD-1 are also associated with T cell inhibition (101). The strong CD4+ T cell response during acute HCV infection is associated with virus clearance. The lack of strong CD4+ T cell response during acute infection and the decline of CD4+ T cell response after acute infection are related to chronic progression (102). Regulatory T cells such as CD25+ T cells can inhibit CD8+ cells and cytokines (such as IL-10 and transforming growth factor TGF-β) release to inhibit immune response during chronic HCV infection (103, 104).

HBV and HCV share the same transmission mechanism, thus coinfection of HBV and HCV is common, particularly in high endemic areas where individuals have a high risk of parenteral infection. The prevalence of HBV/HCV coinfection is approximately 5%-20% in HBsAg positive patients and 2%-10% in HCV-positive patients (105). A prevalence of overt HBV coinfection in HCV positive patients was reported at 1.4% in the United States (106).

Both HBV and HCV complete their life cycle in hepatocytes, and HCV core protein strongly inhibits HBV replication during HBV/HCV coinfection (107). A recent study found that HCV core protein inhibits HBV replication by downregulating HBx levels *via* Siah-1-mediated proteasomal degradation during coinfection (108). HCV core gene also inhibits the induction of an immune response to HBsAg. The observed interference effect of the HCV core occurs in the priming stage and is limited to the DNA form of the HBsAg antigen, but not to the protein form (109). HCV plays a dominant role, so high HCV RNA and low HBV DNA levels are observed in most cases with HBV/HCV coinfection. The cure of HCV infection may lead to HBV reactivation, and a meta-analysis showed that the pooled proportion of patients who had HBV reactivation was 24% in patients with chronic HBV infection and 1.4% in those with resolved HBV infection (110). HBV reactivation is the result of the weakening of hepatocyte IFN response after HCV clearance. Higher serum TNF-α at baseline and lower IFN-γ at week 4 were associated with mild clinical reactivation of HBV in HBV/HCV-coinfected patients receiving direct-acting antiviral agents (DAAs) (111). Chemokine ligand CXCL-10 (another name is interferon induced protein-10, IP-10), CCL5 and ALT have predictive value for HBV reactivation after HCV clearance (112). On the other hand, exogenous HBsAg stimulated NKG2D expression on NK cells from CHB patients, which inhibits HCV replication, suggesting that HBsAg may facilitate the clearance of HCV in HBV/HCV-coinfected patients (113).

In HBV/HCV coinfection patients, the HBsAg level is usually lower than that in HBV monoinfection patients, and the decrease of HBsAg production is also related to the increase of CXCL-10 level (114). A 5-year follow-up study in HBV/HCV coinfection patients showed that the cumulative HBsAg seroclearance rate was 30.0%, with 33.1% in the 48-week PEG-IFN plus ribavirin combination therapy group, and 24.3% in the 24-week therapy group (115). DAAs-treated HBV/HCV-coinfected patients had significantly higher rate of HBV seroclearance, particularly among those with low pre-treatment HBsAg titer; on the contrary, those with higher pre-treatment HBsAg titer were at greater risk of HBV reactivation (116).

### HBV/HDV Coinfection

HDV is a defective virus, which relies on HBV for packaging, release and transmission. The global total prevalence of hepatitis D varies greatly in various literatures. The rate of HBsAg positive patients complicated with HDV infection ranges from 4.5% to 13.02% (117–121). This difference may be related with the inconsistent diagnostic criteria of hepatitis D. HBV/HDV coinfection can cause the most severe viral hepatitis.

Due to severe inflammation and necrosis of hepatic lobules, liver biopsy showed that the degree of liver injury during coinfection was almost twice that of HBV or HCV monoinfection (122). Due to HDV-induced interferon response (123), pronounced induction of innate immune responses (such as elevated cytokine levels of ISGs, TGF-β, IFN-γ, IP-10, etc.) may lead to a higher degree of liver inflammation compared with HBV monoinfection, resulting in a more severe infection process (124).

In Huh7 and HEK293 cells, large hepatitis D antigen (L-HDAg) can interfere with TNF-α-NF-κB signal transduction axis (125). L-HDAg can enhance TGF-β-c-Jun induced signal cascade, while TGF-β is the main regulator of liver fibrosis and cirrhosis (126). L-HDAg can also induce oxidative stress and activate NF-κB and signal transducer and activator of transcription-3 (STAT-3), leading to liver cirrhosis and cancer (127). HBsAg may increase the translocation of L-HDAg from nucleus to ER, and the translocation is accompanied by an increase in NF-κB activity (128). Compared with HBV monoinfection, the upregulation of antigen processing mechanism leads to higher efficiency of HBV epitope presentation in HBV/HDV coinfected cells, which can promote the recognition of infected cells by T cells (129).

Although HDV has been shown to inhibit HBV replication in many studies, serum HBsAg levels in patients with HBV/HDV coinfection are higher or equal than those in patients with HBV monoinfection (130, 131). HBV/HDV coinfected sequences exhibited certain unique mutations in HBsAg genes. Some of these mutations affected the generation of proteasomal sites, binding of HBsAg epitopes to MHC-I and -II ligands, and subsequent generation of T- cell epitopes. Selective amplification of these mutations at certain strategic locations might not only enable HBV to counteract the inhibitory effects of HDV on HBV replication, but also facilitate its survival by escaping the immune response (132). The percentage of conserved HBsAg-positions was significantly higher in HBV/HDV coinfection than HBV monoinfection. HDV can constrain HBsAg genetic evolution to preserve its fitness (133).

### HBV/HIV Coinfection

HIV is a non-hepatophilic virus that mainly invades lymphocytes. The human immune function gradually loses and eventually leads to acquired immune deficiency syndrome (AIDS) by HIV infection. Host and virus jointly determine the disease progression after HIV infection, in which the activation level of innate immunity plays an important role (134).

Evolution during primary HIV infection does not require adaptive immune selection (135). It is found that DC, NK cells, macrophages and NKT cells play an important and irreplaceable role in innate immunity in long-term nonprogressors and elite controllers (that means with HIV-1 infection for many years, long-term asymptomatic, normal CD4^+^ T cell count and no antiretroviral therapy) of HIV infection. Other innate immune cells are inefficient or even ineffective.

The progression of HIV infection may be related to the number and phenotypic function of DC. In patients with typical progression of HIV-1 infection, the number and phenotype of DC change with the progression of the disease (136), while DC in elite controllers can enhance and expand the ability to stimulate HIV specific CD8^+^ T cell response by improving the internal immune recognition of HIV infected cells. Type I IFN secreted by DC plays an important role in inducing effective HIV specific CD8^+^ T cell immunity (137).

The dysfunction of DC after HIV infection contributes to the persistence of the virus (138). NK cell activity is in the normal range in long-term nonprogressors, but decreased in patients with disease progression, indicating that NK cell activity is an important factor in controlling the progression of HIV infection (139). The levels of macrophage inflammatory protein (MIP), IP-10, monocyte chemoattractant protein-1 (MCP-1) and TGF decrease in elite controllers of HIV infection (140). Increased expression of CD224 on NKT cells is associated with HIV disease progression (141), and NKT cells in non-progression patients secrete more IFN-γ, IL-2 and TNF-α than those in progressive patients. These cytokines can significantly reduce HIV viral load and maintain a high number of CD4^+^ T cells (142).

In addition to the difference of innate immunity, adaptive immunity, such as CD8^+^ T cell function, has also been enhanced in HIV elite controllers. HIV-1 specific CD8^+^ T cells of elite controllers can reduce HIV-1 replication in infected CD4^+^ T cells by 60%-80%, and can also recognize resting infected CD4^+^ T cells and kill these cells without virus activation (143–146). There are a small amount of HIV-1 specific CD57^+^ CD4^+^ T cells in elite controllers, which may play a direct role in killing virus infected cells and supplement the cytotoxic activity of HIV-1 specific CD8^+^ T cells (147). Elite controls can produce effective anti-HIV-1 antibodies, and the frequency of preserved memory B cells is higher (148, 149).

A meta-analysis showed that the global rate of combined HBV infection in HIV patients was 7.6% (150). HIV coinfection has a negative impact on the progress of HBV infection, which can lead to rapid progression to liver fibrosis and cirrhosis (151). Enhanced production of CXCL10 following coinfection of hepatocytes with both HIV and HBV may contribute to accelerated liver disease in the setting of HIV/HBV coinfection (152). It is known that HBV Pre-S deletion is closely related to HBV-associated terminal liver disease in HBV monoinfection. High-frequency Pre-S quasispecies deletions are predominant in HIV/HBV coinfection patients, providing a reference for the pathogenesis of the accelerated progression of liver disease in HIV/HBV coinfection (153). Even after effective antiretroviral therapy, the chronic immune activation of patients with HIV/HBV coinfection is higher than that of patients with HIV momoinfection. Chronic immune activation may lead to hepatic steatosis and cirrhosis, increase the risk of liver cancer (154). At the same time, the presence of active HBV infection will affect the viral immunological status of patients with HIV/HBV coinfection, which is characterized by the low number of CD4^+^ T cells at the onset and the slow recovery of CD4^+^ T cell count after antiretroviral treatment (155, 156).

HBsAg levels and HBsAg production were significantly higher in untreated HIV/HBV coinfection patients compared to HBV monoinfection patients. The highest HBsAg concentrations were observed in patients with more advanced HIV disease (157). Compared with patients with HBV monoinfection, successful long-term tenofovir dipivoxil (TDF) inclusive ART can increase the HBsAg seroclearance rate in HIV/HBV coinfection patients, reaching 3.2%-36% (158–173) (see **Table 1**). The longer the follow-up time, the higher the HBsAg seroclearance rate. Higher HBsAg seroclearance rate is associated with increased CD4^+^ T cells. The sudden recovery of adaptive immunity causes immune reconstitution inflammatory syndrome, and then accelerates the production of protective antibodies. Therefore, immune reconstitution under antiretroviral therapy may affect the HBsAg serum conversion rate.

**Table 1:** HBsAg seroclearance rate in HBV/HIV coinfection patients with TDF inclusive ART.

Publication year	Country or region of patients	Number of patients included	Main ART drugs	Mean follow-up time or therapy duration time	Number or rate of HBsAg seroclearance
2005 (158)	Germany	31	TDF	48 weeks	1/31 (3.2%)
2007 (159)	France	92	LAM	ART: 65 (1-155) months HARRT: 43 (1-93) months LAM: 36 (1-83) months	5/92 (5.4%)
2010 (160)	Dutch	102	TDF LAM ETV	5 years	10/102 (9.8%)
2012 (161)	Dutch	104	TDF	57 (34-72) months	8/104 (7.7%)
2012 (162)	Austria	110	LAM TDF FTC	5 years	HBeAg+: LAM: 8% TDF: 25% TDF+FTC: 27% HBeAg-: LAM: 11% TDF: 27% TDF+FTC: 36%
2013 (163)	Zambia South Africa	92	TDF LAM	12 months	LAM: 4/20 (20%)* TDF:3/17 (17.6%)*
2013 (164)	Thailand	47	LAM FTC TDF	168 weeks	6/47 (12.7%)
2014 (165)	France	111	TDF LAM FTC	74.7 (33.7-94.7) months	No detail data
2015 (166)	USA	99	TDF	5 years	18/99 (18.1%)
2015 (167)	Austria	111	TDF	74.2 (33.1-94.7) months	4/111 (3.6%)
2019 (168)	Taiwan, China	366	TDF LAM	5 years	15/366 (4.1%)
2020 (169)	Zambia	284	TDF	2 years	29/284 (10.2%)
2020 (170)	Australia Thailand	92	TDF	5 years	11/92 (12.0%) 11/72 (15.3%)^#^
2020 (171)	Germany	359	TDF TAF	11 years	66/359 (18.3%)
2021 (172)	France	165	TDF	15 years	13/165 (7.8%)
2022 (173)	USA	88	TDF FTC LAM	144 weeks	TDF+FTC: 30% FTC or LAM: 10%

TDF, tenofovir dipivoxil; FTC, emtricitabine; LAM, lamivudine; ETV, entecavir; TAF, tenofovir alafenamide; ART, antiretroviral therapy; HARRT, highly active antiretroviral therapy

*Because stored samples were unsuitable or not available, they only calculated documented data.

^#^The data of 72 patients was available to year 5.

## Conclusions

The pathogenesis of chronic hepatitis B is mainly related with immune mechanisms. The functional cure of hepatitis B with HBsAg seroclearance as the therapeutic target mainly depends on the immune response. When coinfected with HBV and other viruses, the body immune state becomes more complicated. In some cases, coinfection can improve the seroclearance rate of HBsAg. The specific mechanism needs to be further elaborated for better guiding the clinical application.

## Author Contributions

ML, LZ, and YX contributed to study concept and design. SW, WY, YG, WD, XB, YJL, LY, YL, RL, MC, GS, and LH collected and sorted out literatures. SW and LZ drew pictures. SW, WY, and YG wrote the first draft. ML and YX edited the English version. YX approved the submitted version after modification. All authors contributed to the article and approved the submitted version.

## Funding

This project was supported by National Science and Technology Major Project of China (No. 2017ZX10201201-001-006 and 2017ZX10201201-002-006, and 2018ZX10715-005-003-005), the Beijing Hospitals Authority Clinical medicine Development of Special Funding Support (No. XMLX 201706 and XMLX 202127), the Digestive Medical Coordinated Development Center of Beijing Hospitals Authority (No. XXZ0302 and XXT28), Beijing Science and Technology Commission (No. D161100002716002), Special Public Health Project for Health Development in Capital (2021-1G-4061 and 2022-1-2172), and Beijing Municipal Science and Technology Commission (No. Z151100004015122).

## Conflict of Interest

The authors declare that the research was conducted in the absence of any commercial or financial relationships that could be construed as a potential conflict of interest.

## Publisher’s Note

All claims expressed in this article are solely those of the authors and do not necessarily represent those of their affiliated organizations, or those of the publisher, the editors and the reviewers. Any product that may be evaluated in this article, or claim that may be made by its manufacturer, is not guaranteed or endorsed by the publisher.
